# Nanobody-Nanoluciferase Fusion Protein-Enabled Immunoassay for Ochratoxin A in Coffee with Enhanced Specificity and Sensitivity

**DOI:** 10.3390/toxins14100713

**Published:** 2022-10-19

**Authors:** Kunlu Bao, Xing Liu, Yujing Liao, Zilong Liu, Hongmei Cao, Long Wu, Qi Chen

**Affiliations:** 1 National Engineering Research Center for Bioengineering Drugs and the Technologies, Institute of Translational Medicine, Nanchang University, Nanchang 330031, China; 2 Key Laboratory of Tropical and Vegetables Quality and Safety for State Market Regulation, School of Food Science and Engineering, Hainan University, Haikou 570228, China

**Keywords:** BLEIA, Nb28-Nluc, bioluminescent, ochratoxin A, food safety

## Abstract

Ochratoxin A (OTA), one of the best-known mycotoxins, causes problems concerning food safety with potential toxic effects in humans and animals. So, it is crucial to develop simple and sensitive methods for the detection of OTA. Herein, a nanoluciferase–nanobody fusion protein (Nb28-Nluc)-retaining antibody recognition and enzymatic activity was first prepared, which was then applied as a bifunctional tracer to construct a one-step bioluminescent enzyme-linked immunosorbent assay (BLEIA) for OTA in coffee samples. On the basis of Nb28-Nluc, the BLEIA can be completed with a one-step incubation and detection, with only a substrate replacement from 3,3′,5,5′-tetramethylbenzidine (TMB) to a Nluc assay reagent (Furimazine). Under the optimal experimental conditions, the proposed one-step BLEIA achieved a detection limit of 3.7 ng/mL (IC_10_) within 3 h. Moreover, the BLEIA method showed good repeatability and accuracy in the spike recovery experiments with recoveries of 83.88% to 120.23% and relative standard deviations (RSDs) of 5.2% to 24.7%, respectively. Particularly, the BLEIA displayed superior performances, such as fewer operations and more rapid and sensitive detection as compared with Nb28-based enzyme-linked immunosorbent assay. Therefore, the proposed one-step BLEIA has great potential for the sensitive and accurate screening of OTA in food samples.

## 1. Introduction

Ochratoxin A (OTA), a second metabolite mainly produced by fungi (*Aspergillus* and *Penicillium*) [1], can easily pollute agricultural crops and related products, such as corn, coffee, peanut, rice, maize and even milk [2,3,4,5]. Studies have shown that OTA displays liver and kidney toxicity, carcinogenesis and mutagenic effects on both humans and animals [4,6,7]. OTA has been classified as 2B carcinogen (possibly carcinogenic to humans) by the International Cancer Research Center (ICRC), and constant exposure to OTA will definitely pose high risks on human health. Lemming et al. found that blood and urine OTA was associated with the intake of raisins and coffee by a survey of approximately 3000 school students in Sweden [8].

Coffee is one of the three most widely consumed beverages in the world and also one of the most valuable agricultural products in international trade. In this regard, OTA contamination may cause not only potential public health risks but also the interruption of global agricultural trade and the hinderance of socioeconomic development [9,10]. To avoid the risk of OTA, many organizations regulate the maximum residue levels of OTA strictly and scientifically in food. For instance, the European Food Safety Authority (EFSA) and the China State Administration for Market Regulation (CSAMR) have set the maximum limits of OTA in roasted and instant coffee at 5.0 and 10.0 μg/kg, respectively [11]. To this end, it is an urgent need to develop effective techniques for the detection of OTA.

In the past decades, numerous analytical techniques have been developed to detect OTA, including high performance liquid chromatography (HPLC) [12], liquid chromatography tandem mass spectrometry (LC-MS/MS) [13], enzyme-linked immunosorbent assay (ELISA) [14], lateral flow immunoassay (LFIA) [15] and biosensors [16]. Among these, ELISA is the most commonly used rapid screening method due to its high throughput and simple operations [17]. However, the traditional ELISA procedure requires several repeated cycles of reagent addition, incubation and microplate washing to add new reagents or to remove unbound reagents, resulting in a longer detection time [18]. With the development of gene engineering and antibody engineering, various miniaturized recombinant antibodies have received extensive attention [19,20]. Moreover, the recombinant antibody-enzyme fusion protein, as a novel bifunctional tracer, has shown significant advantages in terms of simplifying the operation process, improving result stability and reducing the detection time in immunoassays [21,22,23,24,25]. As reported, nanoluciferase (Nluc), the smallest commercially available luciferase reporter (19 kDa), can offer the brightest bioluminescence with over a 150-fold enhancement compared with that of traditional luciferase [26,27]. Nluc has been used as an excellent fusion protein partner to develop bifunctional tracers for antibody screening [26], protein–protein interaction analysis [28], disease diagnosis [29], bioluminescence imaging [30] and immunoassay [31,32].

**Figure 1:**
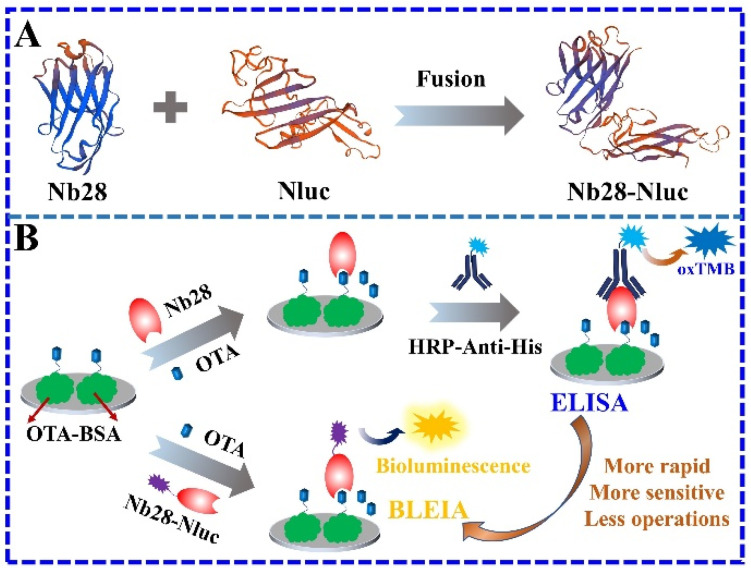
Schematic illustration of the one-step BLEIA based on bifunctional tracer (Nb28-Nluc fusion protein) for the detection of OTA. (**A**): OTA-specific nanobody Nb28 fused with Nluc to prepare bifunctional tracer (Nb28-Nluc); (**B**): Nb28-Nluc bifunctional tracer simplified the operation process of the developed BLEIA.

In this work, on the basis of OTA-specific nanobody Nb28 and Nluc, we first prepared a nanobody/Nluc fusion protein (Nb28-Nluc) by prokaryotic expression in *E. coli* Rosetta (Figure 1A). In order to explore the feasibility of Nb28-Nluc as a novel bifunctional tracer in immunoassay, a bioluminescent enzyme immunoassay (BLEIA) for the sensitive and rapid detection of OTA was further developed (Figure 1B). On the basis of Nb28-Nluc, the detection of OTA can be realized with a one-step of incubation and detection, with only a substrate replacement of 3,3′,5,5′-tetramethylbenzidine (TMB) with furimazine, a luciferase assay reagent. The optimization of experimental parameters, including the concentrations of the coating antigen (OTA-BSA), bifunctional tracer (Nb28-Nluc), salt ions and methanol in the assay buffer and the competitive reaction time were investigated. Under the optimized experimental conditions, the developed BLEIA can complete the detection of OTA in coffee samples within 3 h, demonstrating its great potential for the sensitive and rapid detection of OTA in food samples.

## 2. Materials and Methods

### 2.1. Chemicals and Reagents

Premix Taq enzyme, restriction endonucleases (*Hind* III, *Xho* I) and T4 DNA ligase were purchased from Takara Biotech Co., Ltd. (Dalian, China). The vector extraction kit and PCR product agarose gel purification kit were obtained from Tiangen Biotech Co., Ltd. (Beijing, China). OTA and OTC were purchased from Pribolab Co., Ltd. (Qingdao, China). OTB was obtained from Bioaustralis Co., Ltd. (Smithfield, Australia). Fumonisin B_1_ (FB_1_), Aflatoxin B_1_ (AFB_1_), zearalenone (ZEN) and deoxynivalenol (DON) were purchased from Fermentek Co., Ltd. (Jerusalem, Israel). White 96-well microplates, Ni-NTA sepharose packing and chromatography columns were purchased from Sangon Biotech Co., Ltd. (Shanghai, China). The pNL1.1 vector with the Nluc gene fragment and Nano-Glo^®^ Luciferase Assay Reagent were purchased from Promega Co., Ltd. (Madison, USA). The pET25b vector, *E. coli* (DH5α cells and Rosetta cells) were saved in our laboratory. The recombinant prokaryotic expression vector (pET25b-Nb28) was developed by our previous work [33]. The OTA-BSA-coated antigen was prepared by covalently combining the carboxyl group of OTA with BSA through an EDC-mediated reaction.

Except for the reagents used in HPLC-FLD, which uses a guaranteed reagent, all chemical reagents were analytical grade. An ultra-micro spectrophotometer (Nanodrop 2000) was obtained from Thermo Fisher Scientific Inc. (Waltham, MA, USA) to measure the concentration of nucleic acid and protein. A SP-Max3500FL multifunctional fluorescence microplate reader was purchased from Flash Spectrum Inc. (Shanghai, China) to measure the bioluminescence at 460 nm from the Nluc-catalyzed reaction.

### 2.2. Construction of the pET25b-Nb28-Nluc Expression Vector

The pET25b-Nb28-Nluc recombinant expression plasmid was constructed as follows. First, the paired primers (Table S1) were used to amplify the gene fragments encoding Nluc and Nb28 from the pNL1.1 and pET25b-Nb28 plasmid by PCR, respectively. The amplified gene fragments were first purified by an agarose gel recovery kit and then Nb28 and Nluc gene fragments were assembled by overlap extension polymerase chain reaction (OE-PCR) to produce the Nb-Nluc full-length gene fragments, which encoded Nb28-Nluc fusion protein with a flexible amino acid linker (GGGGS)3 and *Hind* III and *Xho* I restriction sites. After restriction enzyme digestion, the Nb28-Nluc gene fragments were gel purified and ligated with the similarly digested pET-25b at a 3:1 molar ratio. The ligated recombinant expression vectors were transformed into *E. coli* DH5α chemically competent cells through a brief heat treatment (42 °C, 90 s). Then, the transformed bacteria were cultured overnight on LB agar plates containing 100 μg/mL ampicillin (Amp) at 37 °C. The random selection of several Amp-positive bacteria was followed by DNA sequencing identification using paired universal primers (T7 and T7-Ter).

### 2.3. Expression and Identification of the Nb28-Nluc Fusion Protein

The Nb28-Nluc fusion protein was produced by autoinduction expression, according to our previous work [34]. First, the recombinant expression plasmid pET25b-Nb28-Nluc was transformed into chemically competent cells (*E. coli* Rosetta) by heat treatment at 42 °C for 90 s. Then, the transformed *E. coli* Rosetta cells were cultured in 300 mL of autoinduction medium at 37 °C with shock (180 rpm) until the OD_600_ reached a value of 0.6. Then, the culture conditions were changed to 250 rpm and 25°C for protein autoinduction expression. The induced *E. coli* Rosetta bacteria were harvested by centrifugation at 4 °C with 8000× *g* for 15 min, then the cells were resuspended with one-fifth the volume of medium (60 mL) in equilibration buffer (8 mM Na_2_HPO_4_, 2 mM KH_2_PO_4_, 2.6 mM KCl, 137 mM NaCl, 1 mg/mL lysozyme and 1 mM PMSF) and incubated at 37 °C with a slight shock for lysozyme hydrolysis. Next, the bacteria cell wall was broken by ultrasonication in an ice bath to avoid Nb28-Nluc fusion protein degradation or denaturation. The soluble Nb28-Nluc fusion protein in the supernatant was separated by centrifugation (10,000× *g*, 15 min) at 4 °C, and the supernatant was further removed from impurities by filtration. After that, the Nb28-Nluc fusion protein was purified using Ni-NTA affinity chromatography according to the manufacturer’s instructions. Finally, the purity of Nb28-Nluc fusion protein was analyzed with SDS-PAGE and the concentration was determined by Nanodrop 2000. The purified Nb28-Nluc fusion protein was aliquoted and stored at −20 °C for further use.

### 2.4. Construction of BLEIA Based on Nb28-Nluc Bifunctional Tracer

The BLEIA was constructed based on Nb28-Nluc fusion protein, and the details are presented as follows. Briefly, OTA-BSA-coated antigen was diluted with 10 mM PBS (pH 7.4) and incubated in a white 96-well microplate at 37 °C for 2 h. After removing the coating solution, the microplate was washed three times with PBST (10 mM PBS containing 0.05% Tween 20) and blocked with 3% nonfat milk (300 μL/well) at 37 °C for 1 h. After three more washing steps with PBST, 50 µL of OTA solutions with various concentrations and 50 µL of the diluted Nb28-Nluc fusion protein were added to the microplate and then incubated for 45 min at 37 °C. The microplate was washed three times with PBST, and 100 µL of Nano-Glo^®^ Luciferase assay reagent was added to each well with an incubation of 10 min at 37 °C. Finally, the bioluminescence intensity from the enzyme-catalyzed substrates was measured at 460 nm by a multifunctional fluorescence microplate reader. For the best detection performance of BLEIA, the concentrations of the Nb28-Nluc bifunctional tracer; the OTA-BSA coating antigen; and the competition reaction time, ionic strength and methanol concentration in the assay buffer were optimized, respectively.

### 2.5. Specificity Evaluation of the Developed BLEIA

To analyze the specificity of the proposed BLEIA, the cross-reactivity rate (CR) of the developed BLEIA for the OTA structural analogs (OTC and OTB) and mycotoxins commonly found in food (AFB_1_, ZEN, FB_1_ and DON) were measured. The calculation formula of CR (%) was: CR (%) = [IC_50_ (OTA)/IC_50_ (Mycotoxins)] × 100, where the IC_50_ was defined as the lowest concentration of OTA that reached 50% competition for OTA-BSA/Nb28-Nluc binding.

The matrix effect analysis experiments were carried out to evaluate matrix effect of coffee samples, and the samples were purchased from a local supermarket (Haikou, China), which were identified as being OTA-negative by LC-MS/MS analysis. The matrix effect analysis experiments were conducted using the following protocol. Briefly, 1 g of ground coffee sample was weighed, dissolved in 5 mL of 10 mM PBS containing 50% methanol, mixed and shaken violently and sonicated for 15 min. After centrifugation at a speed of 10,000× *g* for 15 min, the supernatant was filtered through a 0.45 μm filter to further remove impurities. Next, the filtered supernatant was diluted to 1:20, 1:30, 1:40 and 1:50 with the optimized assay buffer. Then, the OTA standards at various concentrations were added to each diluted supernatant. Finally, under the optimized experimental parameters, the proposed BLEIA was used to directly quantify OTA-spiked samples.

### 2.6. Practicability of the Assay

The practicability of the proposed method was estimated by performing BLEIA using OTA-spiked coffee samples. Prior to BLEIA, the pretreatment of coffee samples was performed as mentioned above. The coffee extraction was spiked with OTA standards of different addition levels (1, 2, 3 mg/kg), and the OTA concentrations were measured by the developed BLEIA to assess the practicability.

## 3. Results and Discussion

### 3.1. Expression, Characterisation and Purification of the Nb28-Nluc Fusion Protein

**Figure 2:**
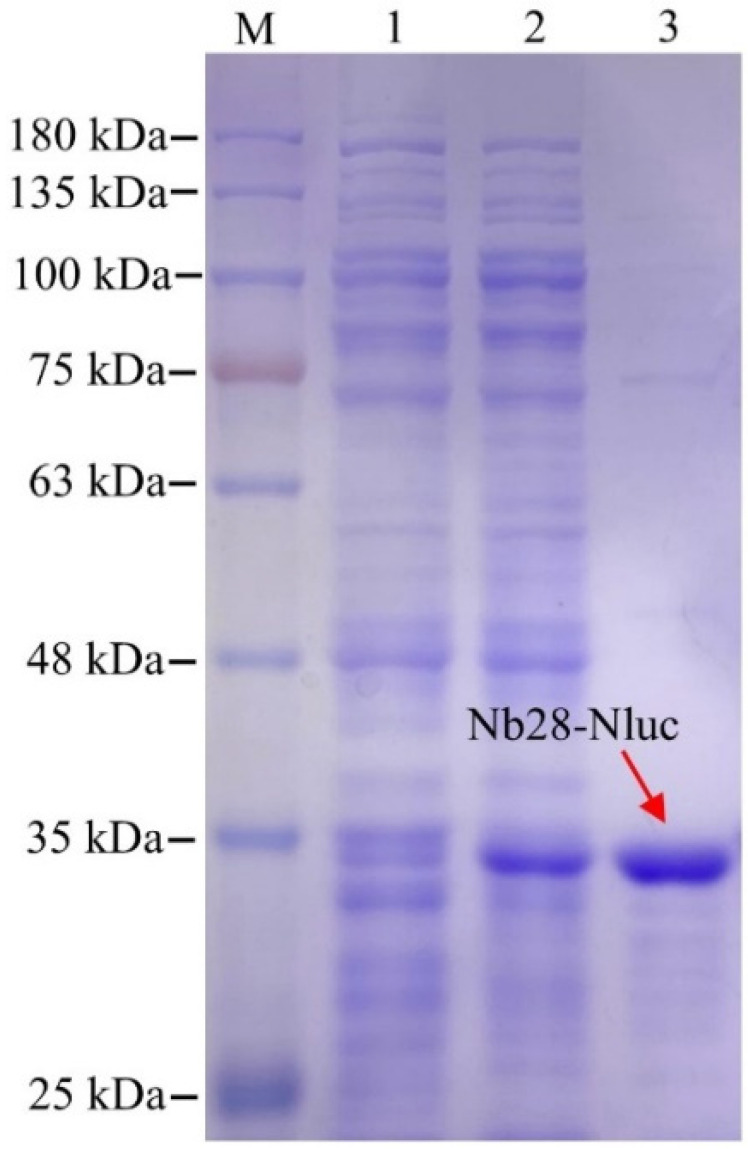
SDS-PAGE analysis of the autoinduction expression of the Nb28-Nluc fusion protein. lane M: the pre-stained protein ladder; lane 1: the total protein of non-induced *E. coli* Rosetta cells; lane 2: the total protein of induced *E. coli* Rosetta cells; lane 3: the purified Nb28-Nluc fusion protein. The red solid arrows point to the Nb28-Nluc fusion protein.

For the expression of the Nb28-Nluc fusion protein, the recombinant plasmid pET25b-Nb28-Nluc was successfully constructed, as shown the Table S1, the Nb28 and Nluc were linked with a flexible amino acid linker (GGGGS)_3_. After autoinduction, ultrasound treatment releases the soluble Nb28-Nluc fusion protein in *E. coli* Rosetta cells into the supernatant, and the target protein can be obtained by Ni-NTA affinity chromatography. Furthermore, 12% SDS-PAGE was used to analyze the expression and purification effects of the Nb28-Nluc fusion protein. As shown in Figure 2, a clear band of about 35 kDa was observed, and the purity of the Nb28-Nluc fusion protein was calculated to be approximately 90%. To evaluate the enzymatic catalytic performance of Nb28-Nluc fusion protein, the enzyme catalytic activity and reaction kinetics were measured. The bioluminescent emission spectra (Figure S1) of Nb28-Nluc in a total volume of 100 μL were measured, and the bioluminescence intensity (at 460 nm) showed a linear relationship with the concentration of Nb28-Nluc fusion protein (Figure S2). The relationship can be described as y = 80,664x − 208.79, with a high correlation coefficient (R^2^ = 0.9923); here, y represents the bioluminescence intensity and x represents the concentration of Nb28-Nluc fusion protein. Moreover, the enzyme catalytic kinetics showed that the bioluminescence intensity had an insignificant change within 60 min (Figure S3). Thus, the purified Nb28-Nluc fusion protein is able to serve as a bifunctional tracer to develop the BLEIA method for OTA detection.

### 3.2. Optimization of BLEIA for OTA Detection

**Figure 3:**
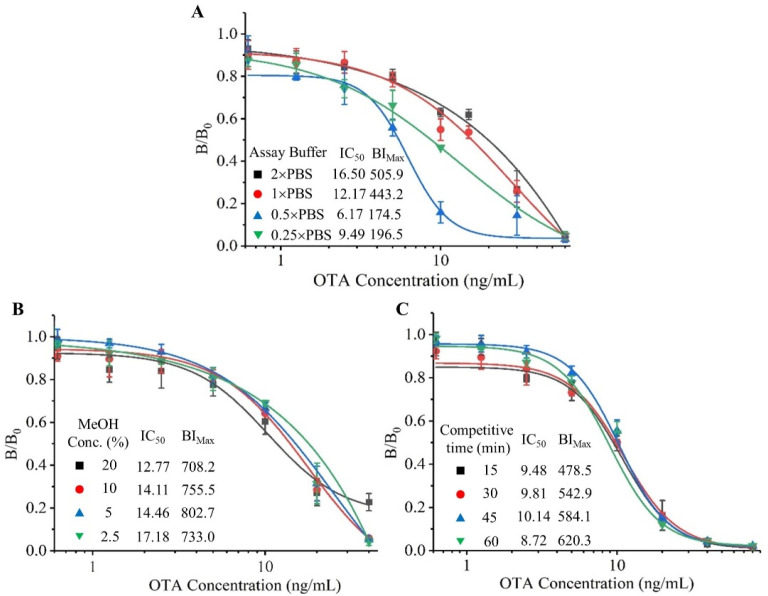
Optimization of the proposed BLEIA experimental parameters. (**A**,**B**): Ionic strength and methanol concentration in the assay buffer; (**C**): Competitive reaction time. The error bars represent the standard deviation of three independent tests.

The experimental parameters were optimized to obtain the best detection performance of the proposed BLEIA, such as the concentrations of the coating antigen (OTA-BSA) and bifunctional tracer (Nb28-Nluc), methanol concentration and ionic strength in the assay buffer, and competitive reaction time. The half inhibitory concentration (IC_50_) and maximum bioluminescence intensity (BI_Max_) were used for the evaluation of the detection effect. As shown in Table S2, using checkerboard titration, the working concentrations of the coated antigen (OTA-BSA, 0.5 μg/mL) and the bifunctional tracer (Nb28-Nluc fusion protein, 0.02 μg/mL) were determined. To assess the influence of ionic strength in the assay buffer, different concentrations of PBS (2.5, 5, 10, and 20 mM) were tested (Figure 3A) and it was evident that the bioluminescence intensity increases significantly with the increase of the ionic strength in the assay buffer. The lowest IC_50_ and the smallest BI_Max_ were observed at 5 mM PBS, which indicated that the lower ionic strength suppresses the bioluminescence signal. To avoid the effect of a signal-to-noise ratio that was too low on the results, 10 mM PBS were selected for further experiments. Since OTA was a highly lipophilic small molecule analyte, methanol was often added to the extraction solution to extract OTA from contaminated food, but methanol often adversely affects immunoassays. To evaluate the influence of methanol on the analysis results, different contents of methanol (20%, 10%, 5% and 2.5%) in the assay buffer were tested. As shown in Figure 3B, the final methanol concentration in the assay buffer was increased from 2.5% to 20% with no apparent change in BI_Max_, which indicated that the proposed BLEIA had strong resistance to methanol interference. Similarly, to shorten the immunoassay time, the competitive reaction time was optimized (Figure 3C). As the time increased from 15 min to 60 min, BI_Max_ increased from 478.5 to 620.3 and 15 min was picked as the optimal competitive reaction time with the lowest IC_50_ value (9.48 ng/mL).

### 3.3. Cross-Reactivity of the Developed BLEIA

Under the optimal conditions, the cross-reactivity (%) of OTA and other four common mycotoxins (AFB_1_, ZEN, DON and FB_1_) and two structural analogs (OTB and OTC) were compared to evaluate the specificity of the developed BLEIA towards OTA. As shown in Figure S4, the cross-reactivity of the proposed BLEIA with the tested common mycotoxins is negligible (<0.5%) and the cross-reactivity of OTB (14.8%) and OTC (3.0%) is low, which was consistent with our previous report [35]. Moreover, these data obviously showed the high selectivity of the developed BLEIA method for the detection of OTA.

### 3.4. Matrix Effect

**Figure 4:**
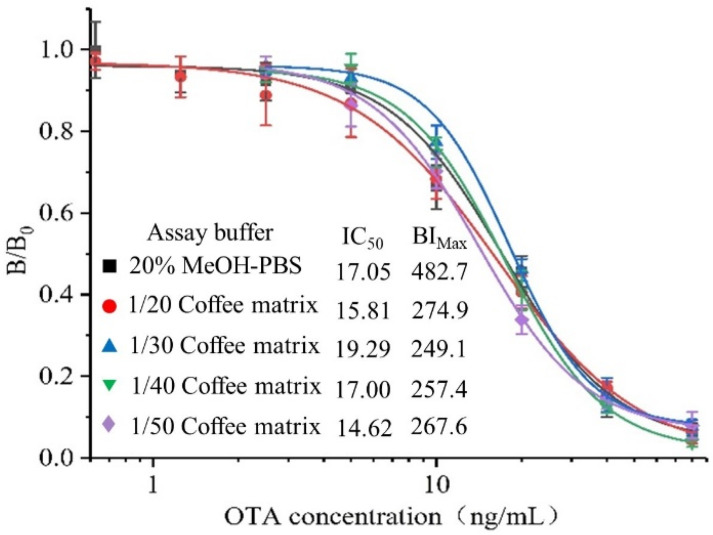
Effect of coffee matrix on the detection performance of the developed BLEIA. Each assay was carried out in triplicate.

Compared with cereals such as wheat, oats and rice, coffee has a more complex matrix and contains proteins, oils, organic acids and pigments (Figure S5), which can significantly affect the sensitivity and accuracy of immunoassays. The dilution method is often used to eliminate the negative effect of this food matrix, while a high dilution ratio may lead to reduced sensitivity. Four standard inhibition curves generated in different coffee extract dilutions (20, 30, 40 and 50 with 20% methanol-10 mM PBS) were compared to evaluate the effect of coffee matrix. The variations in BI_Max_ and IC_50_ of the immunoassay with serially diluted sample matrix extracts are shown in Figure 4. When the coffee sample was diluted 20 times, the corresponding standard curve was the most consistent with the curve generated in the optimized assay buffer without coffee extract. In addition, the matrix effect can not be completely eliminated even if it was diluted 50 times because BI_Max_ is still subject to the downward trend. Taking the sensitivity into account, the coffee sample was diluted 20 times to reduce the negative effect of food matrix for real coffee sample analysis.

### 3.5. Practicability of the Developed BLEIA

The practicability of the developed BLEIA was further evaluated using OTA-spiked coffee samples. Prior to BLEIA, a series of OTA concentrations (1, 2 and 3 mg/kg of samples) were spiked to the OTA-negative coffee samples. After that, the extracted supernatant was diluted with 20% methanol-10 mM PBS at a ratio of 1:20 to minimize the food matrix effect, and then the solutions were applied to BLEIA detection. The intra- and inter-assay recoveries and their relative standard deviations (RSDs) were discussed to evaluate the accuracy and repeatability of the developed BLEIA. As shown in Table 1, the average recoveries of the intra-assay ranged from 88.41% to 110.08% with RSDs of 5.2% to 15.9% and those of inter-assay ranged from 83.88% to 120.23% with RSDs of 6.9% to 24.7%, which demonstrated the good practicability of the developed BLEIA. To verify the reliability of the developed BLEIA, the spiked coffee samples were also analyzed by HPLC-FLD. As shown in Table 1, a good correlation was obtained between the two methods. These above-mentioned results demonstrated the good practicability of the proposed BLEIA.

## 4. Conclusions

In this work, we prepared a Nb28-Nluc bifunctional fusion protein for the development of a one-step BLEIA for the sensitive and rapid detection of OTA in coffee samples. The newly developed method has the following two main advantages: (i) Nb28-Nluc act as a bifunctional probe for antigen recognition and signal amplification, so the incubation step of the secondary antibody can be omitted and the time from sample processing to data analysis can be shortened to less than 3 h, and (ii) the bioluminescence signal can eliminate the interference of the coffee extract color to some extent when compared to colorimetric method. Generally, the BLEIA method can achieve the detection limit of 3.7 ng/mL (IC_10_) with good repeatability (recoveries from 83.88% to 120.23%) and accuracy (RSDs from 5.2% to 24.7%) in the spike recovery experiments and relative standard deviations. Still, the developed BLEIA may suffer from limitations, such as the high dilution of the coffee sample (100×), which can be attributed to the strong impact of the complex coffee matrix for immunoassays. This shows that it is difficult to meet the needs of detecting OTA with high-sensitivity in coffee samples by simple dilution, and the pretreatment of OTA-contaminated coffee samples by affinity column or ion exchange column may be an indispensable step. Moreover, it has been demonstrated to be an efficient tool for the detection of OTA in coffee samples. Additionally, the bifunctional fusion protein as a robust immunochemical reagent has great potential in the detection of other toxic small molecules.

## Figures and Tables

**Table 1:** Practicality of the developed BLEIA.

Spiked OTA (mg/kg)	Nb28-Nluc-Based BLEIA				HPLC-FLD		
		Mean ± SD	Recovery (%)	RSD (%)	Mean ± SD	Recovery (%)	RSD (%)
Intra-assay (n = 3)	1	1.10 ± 0.17	110.08%	15.9%	1.01 ± 0.09	101.27%	9.19%
	2	1.98 ± 0.22	98.92%	11.3%	2.03 ± 0.13	101.33%	6.28%
	3	2.65 ± 0.14	88.41%	5.2%	2.68 ± 0.24	89.22%	9.13%
Inter-assay (n = 3)	1	1.20 ± 0.30	120.23%	24.7%	1.00 ± 0.10	99.93%	10.47%
	2	1.85 ± 0.17	92.63%	9.3%	2.03 ± 0.14	101.56%	6.68%
	3	2.52 ± 0.17	83.88%	6.9%	2.82 ± 0.18	94.11%	6.42%

## Data Availability

The data presented in this study are available on request from the corresponding author.

## Supplementary Materials

The following supporting information can be downloaded at: https://www.mdpi.com/article/10.3390/toxins14100713/s1, Figure S1: The bioluminescent emission spectra of Nb28-Nluc fusion protein; Figure S2: The enzyme catalytic activity analysis of Nb28-Nluc fusion protein; Figure S3: The enzyme catalytic kinetic analysis of Nb28-Nluc fusion protein; Figure S4: Cross-reactivity of the Nb28-Nluc fusion protein based BLEIA with common mycotoxins; Figure S5: The coffee extract of different dilutions with 20% methanol-10 mM PBS; Table S1: The primers and amino acid sequence of Nb28-Nluc fusion protein; Table S2: Optimization of OTA-BSA and Nb28-Nluc concentration by checkerboard titration.
