# Supplementary material for: Nanobody-Nanoluciferase Fusion Protein-Enabled Immunoassay for Ochratoxin A in Coffee with Enhanced Specificity and Sensitivity
Source: Toxins (Basel). 2022 Oct 19;14(10):713. doi: 10.3390/toxins14100713 (PMC9609117; doi:10.3390/toxins14100713)
Supplement: Supplementary file 1 [file toxins-14-00713-s001.zip › toxins-1953363-supplementary.pdf]

toxins

Supplementary material

Nanobody-Nanoluciferase Fusion Protein-Enabled Immunoassay for Ochratoxin A in Coffee with Enhanced Specificity and Sensitivity

Kunlu Bao [1,†], Xing Liu [1,†], Yujing Liao [1], Zilong Liu [1], Hongmei Cao [1], Long Wu [1] and Qi Chen [1,2,*]

1 Key Laboratory of Tropical and Vegetables Quality and Safety for State Market Regulation, School of Food Science and Engineering, Hainan University, Haikou 570228, China
2 National Engineering Research Center for Bioengineering Drugs and the Technologies, Institute of Translational Medicine, Nanchang University, Nanchang 330031, China
* Correspondence: qichen@ncu.edu.cn
† These authors contributed equally to this work.

Table of contents

Figure S1……..…………………..…………………..……………….…………….………….…………s-2

Figure S2……..…………………..…………………..……………….…………….………….…………s-3

Figure S3……..…………………..…………………..……………….…………….………….…………s-4

Figure S4……..…………………..…………………..……………….…………….………….…………s-5

Figure S5……..…………………..…………………..……………….…………….………….…………s-6

Table S1……..…………………..…………………..……………….…………...………….……………s-7

Table S2……..…………………..…………………..…………………..…………………..………… s-8

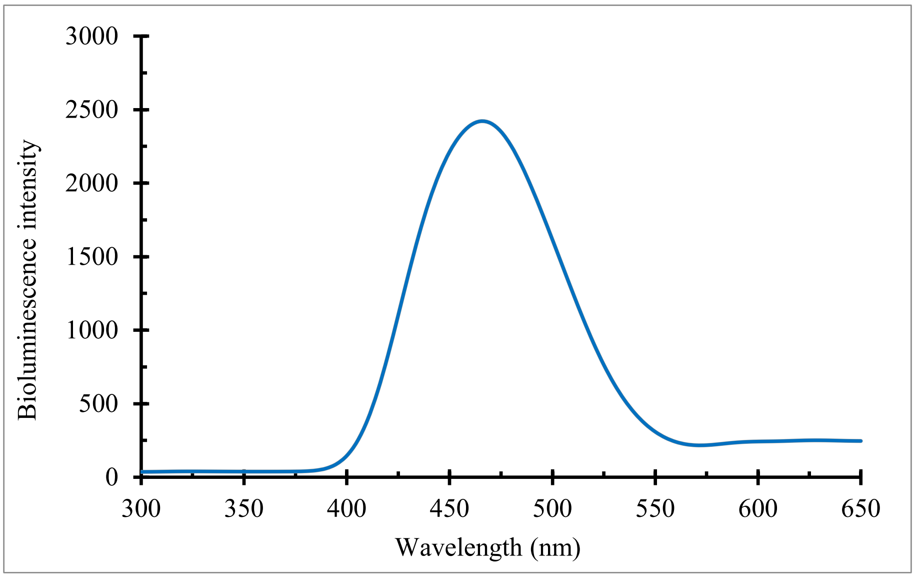

Figure.S1. The bioluminescent emission spectra of Nb28-Nluc fusion protein.

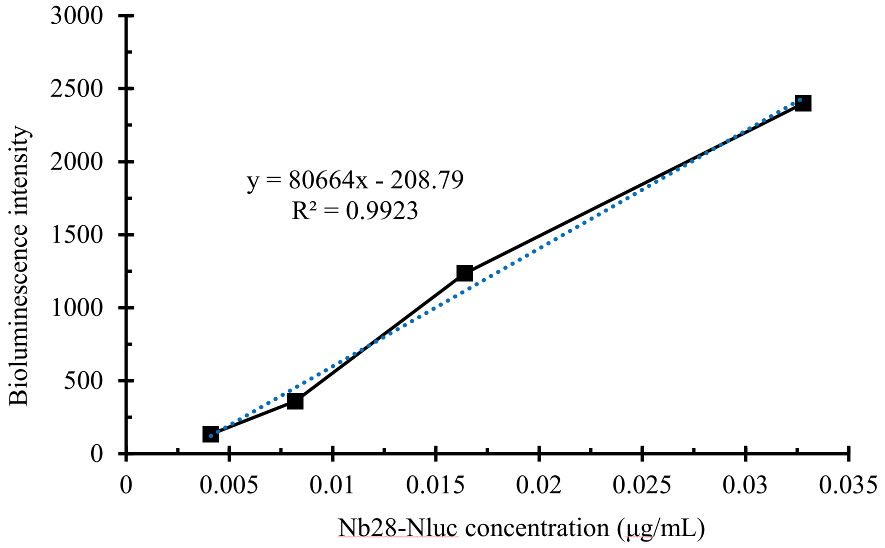

Figure.S2. The enzyme catalytic activity analysis of Nb28-Nluc fusion protein.

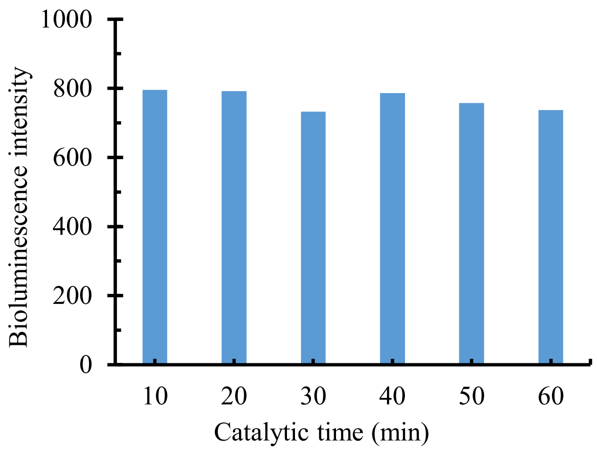

Figure.S3. The enzyme catalytic kinetic analysis of Nb28-Nluc fusion protein.

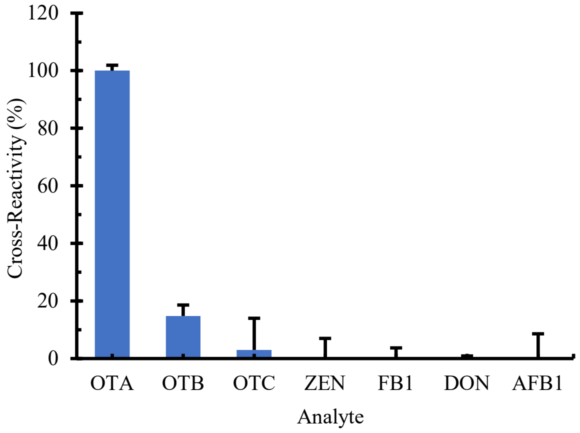

Figure. S4. Cross-reactivity of the Nb28-Nluc fusion protein based BLEIA with common mycotoxins

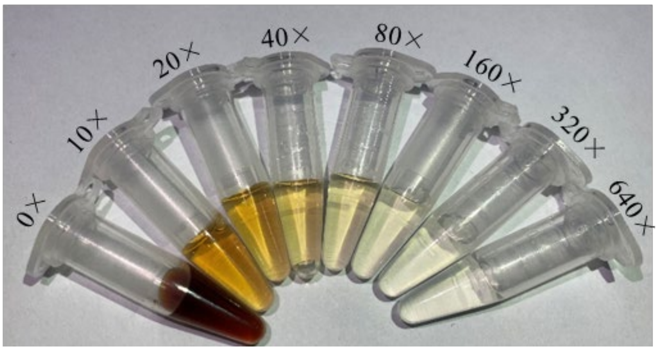

Figure. S5. The coffee extract of different dilutions with 20% methanol-10 mM PBS.

Table S1. The primers and amino acid sequence of Nb28-Nluc fusion protein.

Name	Primers	Sequence
Nb28	VN-VF	5-CCGCTCGAGATGGCCATGGCCCAGTTGC-3
	VN-VR	5-CCGCCAGAGCCACCTCCGCCTGAACCGCCTCCTCCTTGTGGTTTTGGTGTCTTGGGTTC-3
Nluc	VN-NF	5-GTTCAGGCGGAGGTGGCTCTGGCGGTGGCGGATCCATGGTCTTCACACTCGAAGATTTCG-3
	VN-NR	5-CCCAAGCTTCGCCAGAATGCGTTCGCAC-3
Nb28-Nluc fusion protein: Nb28 sequence labeled in blue and Nluc labeled in yellow		QLQLVESGGQLVQAGGSLRLSCAASGSTVGVNAMDMGWYRQAPGKQRELVAAIINGGGDTNLADSVKGRFTISRDGAKRTLYLQMNSLKPEDTAVYYCYVRSGVGLVYWGQGTQVTVSSEPKTPKPQGGGGSGGGGSGGGGSMVFTLEDFVGDWRQTAGYNLDQVLEQGGVSSLFQNLGVSVTPIQRIVLSGENGLKIDIHVIIPYEGLSGDQMGQIEKIFKVVYPVDDHHFKVILHYGTLVIDGVTPNMIDYFGRPYEGIAVFDGKKITVTGTLWNGNKIIDERLINPDGSLLFRVTINGVTGWRLCERILAKLAAALEIKRASQPELAPEDPEDVEHHHHHH

Table S2. Optimization of OTA-BSA and Nb28-Nluc concentration by checkerboard titration.

Nb28-Nluc	OTA-BSA（μg/mL）				
（μg/mL）	4	2	1	0.5	0.25
1	660.0805	635.5025	627.2535	582.35	431.6403
0.5	388.5102	382.799	369.2238	308.6288	277.559
0.25	281.5274	283.1213	247.0677	243.9467	209.2187
0.125	172.7527	180.6345	173.6411	149.8517	143.1981
0.0625	112.1449	101.7076	101.9185	107.2707	82.8427
0.03125	82.7355	66.7927	58.9939	59.8697	53.5302
0.015625	52.2027	50.8871	52.8408	37.8321	44.6096
Control	22.3935	24.5241	26.7209	28.0012	27.0212
